# Elderly Stroke Rehabilitation: Overcoming the Complications and Its Associated Challenges

**DOI:** 10.1155/2018/9853837

**Published:** 2018-06-27

**Authors:** Siew Kwaon Lui, Minh Ha Nguyen

**Affiliations:** ^1^Department of Rehabilitation Medicine, Singapore General Hospital, 20 College Road, Academia Level 4, Singapore 169856; ^2^Department of Geriatric Medicine, Singapore General Hospital, 20 College Road, Academia Level 3, Singapore 169856

## Abstract

There have been many advances in management of cerebrovascular diseases. However, stroke is still one of the leading causes of disabilities and mortality worldwide with significant socioeconomic burden. This review summarizes the consequences of stroke in the elderly, predictors of stroke rehabilitation outcomes, role of rehabilitation in neuronal recovery, importance of stroke rehabilitation units, and types of rehabilitation resources and services available in Singapore. We also present the challenges faced by the elderly stroke survivors in the local setting and propose strategies to overcome the barriers to rehabilitation in this aging population.

## 1. Background

Despite advances in modern medicine, medications, and medical technology, stroke diseases impose a substantial mortality and morbidity risk to the individual with increased economic burden to the society. Globally, stroke is the second leading cause of death after ischemic heart disease, with approximately 6.7 million stroke deaths in 2015 [1]. In Singapore, despite decreasing trend, cerebrovascular diseases are still the fourth leading cause of death, with a prevalence of 6.6% in 2016 [2]. As the population rapidly ages, the burden of stroke is expected to increase significantly, posing challenges to limited healthcare resources.

As such, there is an urgent need to develop an optimal stroke disease management plan, incorporating a comprehensive stroke rehabilitation program.

## 2. Consequences of Stroke in Elderly Stroke Survivors

The incidence of stroke disease increases with age, in both men and women with approximately 50% of all strokes occurring in people over age 75 and 30% over age 85 [1, 3, 4]. Stroke is among the top leading causes of disability and reduced quality of life [5]. Elderly patients are at higher risk of mortality, poorer functional outcomes, prolonged length of hospital stay, and institutionalization [6].

Motor impairment is the most common deficit after stroke, which either happens as a direct consequence of the lack of signal transmission from cerebral cortex or as a slowly accumulating process of the cerebral injuries or muscle atrophy due to learned disuse [7, 8]. Divani et al. reported the risk of falling and fall-related injuries were higher in stroke elders [9]. Risk factors associated with increased fall risks in stroke survivors include poor general health, time from first stroke, psychiatric problems, urinary incontinence, pain, motor impairment, and a history of recurrent falls [9]. Risk factors associated with fall-related injuries are female gender, poor general health, past injury from fall, psychiatric problems, urinary incontinence, impaired hearing, pain, motor impairment, and presence of multiple strokes [9]. Motor function deficits, increased fall risks, and fall-related injuries can significantly affect the patients' mobility, and their daily living activities which limit their participation in social events and other professional activities.

Poststroke cognitive impairment is common and can affect up to one-third of stroke survivors [10, 11]. However, subtle cognitive impairment may not appear apparent, especially when the stroke survivor seems to have recovered functionally in other aspects [10, 11]. In most cases, these deficits are persistent and usually have progressively worsened [12]. Poststroke cognitive impairment is also more common in those with recurrent strokes [13]. It often coexists with other neuropsychological problems including language disorders, fatigue, depression, and apathy [13]. The mechanisms of poststroke cognitive impairment could be either directly due to cerebral vascular injury or indirectly due to an associated asymptomatic Alzheimer pathology or white matter changes from small vessel disease [14]. Factors independently associated with dementia in stroke survivors include atrial fibrillation, previous stroke, myocardial infarction, hypertension, diabetes mellitus, and previous transient ischemic attack [15]. The combined motor and cognitive impairments significantly increase risks of long term functional disability and increase healthcare cost as reflected by an increase in hospital readmission rates and mortality rates [16].

Bladder and bowel dysfunction are common and cause significant distress to stroke survivors. Poststroke urinary incontinence or retention has been shown to affect about 30% of stroke survivors [17]. Urinary incontinence is an important marker of stroke severity and has been linked with functional dependency, increased risk of institutionalization, and mortality [17]. Risk factors for poststroke urinary retention include cognitive impairment, diabetes mellitus, aphasia, poor functional status on admission, and urinary tract infection [18]. Common gastrointestinal symptoms after stroke include dysphagia, heartburn, abdominal pain, fecal incontinence, bleeding gastrointestinal tract, and constipation [19]. Among these, constipation is the most common bowel dysfunction with the incidence ranging from 29% to 79% in stroke survivors and more prevalent in hemorrhagic stroke patients [20]. Although fecal incontinence is less common with a prevalence of 11% at 1 year after stroke, it is associated with increased risk of nursing home admission and 1-year mortality rate [21].

Infection is a serious complication after a stroke despite optimal management. The reported prevalence of poststroke infection ranges from 5% to 65%, depending on the study population, study design, and the definition of infection [22]. Mortality rate is higher in stroke patients with any type of infection, particularly higher in patients with pneumonia and patients with urinary tract infection [23]. Among the survivors, stroke-associated infection is also an independent risk factor for poor outcome at discharge and at 1 year [23]. The association between poststroke infection and poor outcome is likely related to a delay in rehabilitation due to prolonged hospital stay and immobilization as well as general frailty [22]. More importantly, evidence from experimental studies suggests that infection also promotes antigen presentation and autoimmunity against the brain which worsens the outcome [24].

Following a stroke, patients may have impaired mobility which predisposes them to pressure sores and deep vein thrombosis (DVT). Pressure ulcer results from an imbalance between external mechanical forces acting on skin and soft tissue and the internal susceptibility of skin and its underlying soft tissue to injury. Pressure ulcer is associated with increased poststroke mortality in both genders and patients aged 60 years or older [25]. Stroke patients also have an increased risk of developing deep DVT and pulmonary embolism due to immobility and raised prothrombotic activity [26]. The major risk factors of poststroke DVT include advanced age, male gender, congestive heart failure, malignancy, and fluid and electrolyte disorders [27, 28].

Pain is a frequent but often neglected complication of stroke [29, 30]. It can happen immediately, weeks, or months after a stroke event and can span a spectrum from irritating headache to debilitating limb pain secondary to complex regional pain syndrome, spasticity or joint subluxation, and /or contractures [29]. Pain, together with depression and fatigue, is associated with increased risk of cognitive impairment, functional dependence, and reduced quality of life in stroke survivors [30, 31]. Reported risk factors for the development of poststroke pain include female gender, older age at stroke onset, history of alcohol use and depression, anatomical location of stroke and presence of clinical features such as spasticity, reduced upper extremity movement, and sensory deficits [32].

## 3. Predictors of Good Rehabilitation Outcome in Elderly Stroke Survivors

Due to the medical complications after stroke, many patients are markedly functionally disabled when they are discharged from acute care. Functional recovery is based on the restitution of brain tissue and on the relearning of and compensation for lost functions [33]. Therefore, understanding and identification of predictors of good rehabilitation outcomes in addition to institution of early rehabilitation are essential in the recovery phase after an acute stroke event.

There are several commonly used tools for measurement of rehabilitation outcomes in stroke patients, including Functional Independence Measure (FIM), Modified Rankin Scale (mRS), and the Barthel Index (BI) [34]. The FIM is the most sensitive and has been widely accepted with good validity and reliability in assessment of the patient's degree of disability and burden of care [34]. It consists of 18 items, 13 items on motor disability, and 5 items on cognitive disability. The FIM is commonly performed on admission and at discharge, with the score range from 18 to 126. Similarly, the BI is a tool used to measure functional ability, consisting of 10 items on mobility, activity of daily living (ADL), bowel, and bladder function. Its scores range from 0 to 100, with a higher score indicating higher functional ability. On the other hand, the mRS is a scale from 0 to 6 that measures the level of a patient's disability.

Age has been well established as a strong predictor of functional outcome and discharge destination in stroke patients in multiple studies across the world in both young and elderly stroke survivors [35–39]. A large community-based cohort study in Denmark reported more than 58% of the very elderly (85 years old and above) were discharged to nursing homes or died during hospital stay poststroke [40]. In a multicenter prospective cohort study of over 300 patients of at least 75 years of age with a first stroke, age was both significantly related to low FIM score upon discharge and independently and inversely related to rehabilitation efficacy (Montebello Rehabilitation Factor Score) [36]. Despite the likelihood of higher comorbidities in older patients, a multicenter cohort study showed that rehabilitation outcomes of elderly patients admitted into skilled nursing facilities (SNFs) were not associated with multimorbidity [41].

Cognitive impairment which occurs either as a prestroke condition or a poststroke is often significantly correlated with reduced functional gains and poor rehabilitation outcomes in elderly patients. A local study by Kong et al. showed that 45% of elderly stroke patients (≥75 years old) admitted to a rehabilitation facility had cognitive impairment and cognition scores strongly predicted functional outcomes [42]. Studies reported evidence of significant impairment of basic and instrumental ADLs in poststroke cognitively impaired elderly survivors [43, 44]. Another study by Pasquini et al. concluded that cognitive impairment (preexisting or new) together with age was the most important predictor of institutionalization 3 years after stroke [45]. Prestroke dementia has been shown to increase risk of 6-month and delayed poststroke mortality [46]. However, elderly stroke patients with cognitive impairments could still benefit from rehabilitation. Rabadi et al. found similar change in total FIM score and FIM efficiency in both cognitively intact and the cognitively impaired groups of stroke patients [47]. Hence, cognitive impairment should be screened for and has to be taken into consideration when rehabilitation goals are formulated and rehabilitation program ought to be individualized according to the stroke survivor's learning ability [48].

ADL dependency on admission, defined as either low FIM score or low BI score, significantly predicts functional dependency outcome in stroke survivors [39, 43, 49, 50]. Elderly stroke patients with poorer preadmission functional status also have longer length of stay and are less likely discharged to an independent or assisted living situation [39, 50, 51]. Similarly, stroke severity, measured by National Institute of Health Stroke Scale, is also another important rehabilitation outcome predictor [49–51]. Furthermore, a recent review by Lazar et al. revealed that aphasia arising from stroke was associated with worse outcomes in both the acute and chronic stroke periods with poorer functional recovery and increased length of rehabilitation and mortality risk [52].

Urinary incontinence is predictive of poor stroke outcome [53]. Mortality at 6 months has been shown to increase in stroke patients with initial urinary incontinence [53, 54]. Ween et al. reported that 64% of incontinent poststroke patients were discharged to nursing homes compared to 18% for continent poststroke patients [55]. The link between urinary incontinence and poor outcomes could be related to incontinence associated with severe hemiparesis, larger stroke lesions, stroke lesion location, and a disruption of the neuromicturition pathways [55–58].

## 4. Role of Rehabilitation Process in Neuronal Recovery

Rehabilitation aims to enhance and augment natural mechanisms of recovery. At the time of ischemic injury, immediate mechanisms of repair are initiated, which include resolution of poststroke edema, variation of function, and reversal of diaschisis. Vicariation refers to neighboring tissues taking over a function lost by the stroke-affected tissue [59]. Diaschisis is based on the mechanism of reduction in metabolism and blood flow of intact brain regions which are distant away from the ischemic core but are still functionally and structurally connected with the ischemic core. It is thought that at least some of the improvement observed after a stroke could be due to the reversal of diaschisis [60, 61]. Such processes lead to “unmasking” of latent networks which can be as rapid as several hours within ischemic injury [62].

Evidence suggests that, within days of stroke, the injured brain has the ability for limited neuronal regeneration by angiogenesis and is coupled with neurogenesis. The ability to self-repair has been shown to happen in aged brains [63]. The repair processes are initially intense and then slow down. Most of the spontaneous stroke recovery occurs in the first 3-6 months after the acute neurological event [64–66]. Generally, patients make 70% of their recovery in the first 3 months after a stroke [67–71]. Despite variations in therapy, such observations of proportional recovery have remained consistent which means that a minimum amount of spontaneous activity and therapy is enough for proportional recovery to happen [72]. An exception to this proportional recovery rule includes damage to the corticospinal tract which results in poorer recovery from impairment [69, 73].

In order to achieve a greater proportion of recovery, a much higher intensity of therapy has to be considered [72]. Greater intensity of stroke rehabilitation has been associated with improved outcomes [74–76]. Skill learning and active participation help to promote plasticity and network activation in stroke recovery [77, 78]. Motor retraining not only enables somatotopic reorganization to happen in perilesional areas and in distant areas connected to the infarct site but also negate the inhibitory effects of myelin associated proteins and ephrins which suppress axonal sprouting [79, 80]. An “enriched environment” in addition to motor retraining has been shown to facilitate motor recovery and neural plasticity in animal studies due to the numerous associated cellular and molecular effects [81–84]. Rehabilitation facilities are ideal enriched environments as they are often situated in stimulating and specialized centers managed by a multidisciplinary team of medical professionals.

## 5. Stroke Rehabilitation Units and Practitioners Involved

Several guidelines recommend all patients admitted with an acute stroke should receive an assessment by a rehabilitation professional [85, 86]. Specialized stroke rehabilitation units have been shown to improve functional outcomes, decrease mortality and reduce length of hospital stay in moderate to severe stroke patients [87]. Combining an enriched environment with skill retraining, stroke rehabilitation units are made up of a multidisciplinary team of medical professionals who offer realistic goal setting and engage in multimodal disability and impairment assessment, medical management, and functional training. The team consists of rehabilitation nurses, occupational therapists, physiotherapists, and speech therapists under the leadership of physicians specialized in rehabilitation medicine. The work of these groups is further supported by dieticians, neuropsychologists, social workers, and recreational therapists such as music therapists. The rehabilitation team addresses the many challenges stroke patients could face such as sensorimotor and balance impairments, dysphagia, cognitive-communication impairments, mood disorders, visual and hearing impairments, and hemispatial neglect. Regular multidisciplinary meetings are conducted to discuss the rehabilitation goals, rehabilitation intervention, functional improvement, discharge planning, and arrangement of outpatient rehabilitation. These structured meetings have been shown to improve functional outcomes [88, 89]. Such collaborative teamwork involves communication among the team members, working towards a common goal and accepting responsibility as a group for the final outcome of the patients [90, 91]. Recommended realistic goals are also planned together with the patients and their caregivers to prepare them for a smooth transition to outpatient rehabilitation and discharge destination with the eventual aim to achieve maximum independence as possible [92].

The hours of therapy vary across different inpatient rehabilitation settings. Generally, most guidelines advocate minimum 45 minutes of each relevant therapy for at least 5 days a week [85, 86, 93]. In United States, inpatient rehabilitation facilities (IRFs) are mandated to provide at least 3 hours of therapy per day for minimum 5 days in a week. Rehabilitation in an IRF improves functional outcomes, independence, and mortality compared to a SNF (subacute rehabilitation), given the interprofessional team of providers, advanced treatment strategies, and the requirement that patients participate in therapy at least three hours daily [86]. Patient's ability to tolerate such level of intensity has to be taken into account when considered for an acute intensive inpatient rehabilitation placement. When the stroke patient is admitted to inpatient rehabilitation, the rehabilitation team would assess the patient and determine an individualized rehabilitation program of suitable intensity and duration to suit the needs for favorable stroke recovery [85].

It is generally recommended to commence stroke rehabilitation as soon as patients are medically stable, to maximize their functional gains and to take advantage of the period of early stroke recovery [85]. However, caution and individualized clinical judgement are indicated especially in older patients and patients with intracerebral hemorrhage [94]. The large multicenter AVERT trial showed that very early, more frequent, and increased dose of mobilization (VEM) intervention reduced the odds of a favorable outcome at 3 months after stroke when compared with usual care (UC) group [95]. However, the median time to first mobilization in both groups was within 24 hours (22.4 hours in UC group versus 18.5 hours in VEM group) [95]. Further analyses from the AVERT study suggested that shorter but more frequent mobilization early after stroke increased the odds of favorable outcome at 3 months when age and stroke severity were controlled [96]. Earlier access to rehabilitation seems to favor better functional outcomes, shorten length of hospital stay, and increase likelihood of discharge to home [97, 98].

## 6. Transitional Care of Poststroke Survivors

Due to residual functional disability and associated medical complications, poststroke elderly survivors and their caregivers often experience significant physical, mental, and social challenges after being discharged home. In most cases, caregivers are usually poorly understood and ill-prepared for their roles and responsibilities they must face at home [99]. As elderly stroke survivors require substantial care demands at home, their caregivers often feel overwhelmed and exhausted, which eventually lead to depression and deterioration of physical health [99].

Definition of transitional care (TC) is widely accepted as “a set of actions designed to ensure the coordination and continuity of healthcare as patients transfer between different locations or different levels of care within the same location” [100]. TC can happen within same setting (e.g., primary care to specialty care); between different settings (e.g., hospital to subacute care); across health states (e.g., acute care to palliative care), or between providers (e.g., generalist to specialist). The different types of TC models for poststroke patients include hospital-initiated support; home-visiting programs; structured telephone support; outpatient setting-based support; lastly, primary patient and caregiver education. A recent meta-analysis by Wang Y et al. reported insufficient evidence to support the role of TC interventions in reduction of mortality and functional improvement after stroke [101]. However, among all the TC interventions, home-visiting programs which focus on patients and caregivers' needs and preferences in addition to well-established rehabilitation goals via multidisciplinary approach seem to be associated with positive outcomes [101]. More research regarding TC interventions needs to be conducted before any further conclusions can be made.

## 7. Rehabilitation Resources and Services in Singapore in addition to the Challenges Faced

In Singapore, elderly stroke survivors after being medically stabilized at the acute hospitals will be transferred to receive inpatient rehabilitation either at rehabilitation units situated within acute hospitals or in community hospitals which are situated as stand-alone units. When the patients are ready for discharge from inpatient rehabilitation, arrangements are made for them to receive outpatient rehabilitation either at hospital outpatient clinic or at day rehabilitation center. Home therapy can be arranged for patients with difficulties to get out of their house. Government subsidies for day rehabilitation centers are available for patients who satisfy certain financial criteria [102].

However, the compliance rate of our local stroke survivors attending outpatient day rehabilitation has been dismal. Two local studies showed the attendance rates of outpatient rehabilitation at 1 year after discharge from community hospitals were 28% and 4.3%, respectively [102, 103]. Reasons for noncompliance to outpatient rehabilitation revolved around the patient's functional, social, financial, medical, and perceptual factors [103]. Firstly, stroke survivors who require ongoing rehabilitation will likely have difficulties in mobility. They also face challenges in stairs and transportation access. Secondly, some elderly live alone and have no caregiver to assist them to the outpatient rehabilitation center [103, 104]. For those with caregivers, the elderly stroke survivors often do not wish to inconvenience them [103]. Thirdly, financial constraint is also commonly cited by the elderly for noncompliance to poststroke medical care and rehabilitation after hospital discharge [103–107]. Although Singaporean residents are eligible for the public healthcare system with significant subsidies from the government, much of the outpatient rehabilitation cannot be paid for with the use of medical savings account (Medicine) or national medical insurance (Medishield) [103]. For those who are qualified for government medical subsidies, the transportation cost and cumulative cost of multiple sessions of outpatient rehabilitation often put them off from continuing rehabilitation [103]. Fourthly, the elderly stroke survivors often suffer from comorbidities which may limit their ability to fully participate in rehabilitation [103, 108]. Cardiovascular and pulmonary diseases such as ischemic heart disease, congestive heart failure, arrhythmias, and chronic obstructive pulmonary disease which are more common in the elderly can result in reduced activity tolerance and restrict them from fully participating in rehabilitation [108]. Vascular-related cognitive impairment which is more common in older stroke survivors could also pose as a barrier to successful rehabilitation [108]. Lastly, although Singapore is one of the most urbanized, modernized, and prosperous countries in Asia, a strong influence of Eastern culture is still present on the local societal perceptions, especially in the elderly. The elderly in the Asian culture are inclined to rely on their children and would perceive rehabilitation as the equivalent of doing exercises at home without the guidance of a therapist and rehabilitation physician. As such, the patients do not see the need to attend outpatient rehabilitation and follow-up [104].

Home rehabilitation could potentially overcome some of the above challenges associated with outpatient rehabilitation. A local study by Tay et al. found most of the stroke patients in an inpatient rehabilitation unit would consider home rehabilitation program (HRP) [109]. As for the minority who declined HRP, reasons given included financial constraint, unsupportive family members, privacy issues, and preference for a hospital-based rehabilitation [109]. As the cost of each home rehabilitation session in Singapore is at least twice as expensive as each outpatient rehabilitation session, most of the patients would be more inclined to undergo HRP if it is Medisave deductible [109].

## 8. Discussion and Conclusions

With the rapidly aging population, several initiatives have been undertaken by the local government to provide better access for the elderly which could overcome the mobility issues faced by elderly stroke survivors. Examples include installation of ramps and additional lifts at local subway stations, introduction of wheelchair-accessible public buses, lift upgrading program to provide lift access on every level of the public housing blocks, and a heavily subsidized public housing home improvement program which includes ramp installation at the entrances of the housing units with steps. As for those elderly stroke survivors who do not have caregivers to assist them to the outpatient rehabilitation center, we propose implementation of an affordable HRP or low cost telerehabilitation. An ongoing local trial looking at telerehabilitation in the first 3 months after stroke perhaps would shed more light on the potential benefit and cost-effectiveness of telerehabilitation in the Singapore poststroke population [110]. The use of home-based robot therapy (HBRT) could also be considered for those who had difficulty in accessing outpatient rehabilitation. Housley et al. found that HBRT reduced costs and increased access of rehabilitation to stroke survivors [111]. In order to rectify the misconception of rehabilitation is the equivalent of doing exercises at home without the guidance of the rehabilitation team, education on stroke rehabilitation should be provided to all stroke patients and caregivers during the acute admission. It has been shown that the use of evidence-based educational guidelines have helped stroke survivors and their families to better understand the importance of stroke rehabilitation, control their comorbidities and cardiovascular risk factors, and reduce their risk of recurrent strokes [112].

In conclusion, stroke in elderly patients poses a major public health concern, due to its strong association with multiple medical complications, poorer functional outcomes, and substantial healthcare cost. For stroke survivors and their families, a good and comprehensive rehabilitation program is the key to recovery and to enable them to reach their highest level of independence as possible. The success of a stroke rehabilitation unit depends on the effective utilization of its resources and seamless coordination between different healthcare professionals as well as the ongoing support from the caregivers and other community services. Provision of evidence-based and culturally relevant stroke rehabilitation will help to effectively manage limited local healthcare resources and improve quality of life in our aging population.
